# Treatments for Staple Line Leakage after Laparoscopic Sleeve Gastrectomy

**DOI:** 10.3390/jcm12103495

**Published:** 2023-05-16

**Authors:** Takashi Oshiro, Kotaro Wakamatsu, Taiki Nabekura, Yuki Moriyama, Natsumi Kitahara, Kengo Kadoya, Ayami Sato, Tomoaki Kitahara, Tasuku Urita, Yu Sato, Makoto Nagashima, Masaru Tsuchiya, Shinichi Okazumi

**Affiliations:** Department of Surgery, Toho University Sakura Medical Center, Sakura 285-8741, Japan; kotaro.wakamatsu@med.toho-u.ac.jp (K.W.); taiki.nabekura@med.toho-u.ac.jp (T.N.); yuuki.01.moriyama@med.toho-u.ac.jp (Y.M.); natsumi.kitahara@med.toho-u.ac.jp (N.K.); kengo.kadoya@med.toho-u.ac.jp (K.K.); ayami@sakura.med.toho-u.ac.jp (A.S.); kita@sakura.med.toho-u.ac.jp (T.K.); urita-04@sakura.med.toho-u.ac.jp (T.U.); yu.sato@med.toho-u.ac.jp (Y.S.); nagashima@sakura.med.toho-u.ac.jp (M.N.);

**Keywords:** laparoscopic sleeve gastrectomy, sleeve leakage, clip treatment, endoscopic balloon dilation, stent treatment, percutaneous transesophageal gastro-tubing, endoscopic vacuum therapy, revisional surgery, obesity

## Abstract

The number of laparoscopic sleeve gastrectomies (LSGs) performed in patients with obesity who are eligible for bariatric and metabolic surgery is currently much lower in Japan than in other countries. Considering the large number of potential patients with obesity and type 2 diabetes and the unique Japanese national health insurance system that guarantees fair healthcare delivery, there is room to increase the number of LSGs in Japan in the near future. However, strict health insurance regulations may limit access to mandatory devices needed to treat postoperative complications, such as staple line leakage, which can cause severe morbidity and even mortality. Therefore, understanding the pathogenesis and treatment options for this complication is crucial. This article examined the current situation in Japan and its impact on staple line leakage management, including the role of endoscopic treatment in reducing reoperation. The authors suggest increasing education and collaboration between healthcare professionals to optimize management and improve patient outcomes.

## 1. Introduction

Laparoscopic sleeve gastrectomy (LSG) is a seemingly simple surgical procedure that involves the removal of 70% of the greater curvature of the stomach and is the only bariatric and metabolic surgery (BMS) covered by insurance in Japan. LSG has a high success rate in weight loss; it improves metabolic diseases, such as diabetes and is currently the most commonly performed BMS worldwide [1]. According to a recent survey by the Japanese Society for Treatment of Obesity (JSTO), of the 985 cases of BMS performed in 75 facilities nationwide in 2022, 913 (92.7%) were LSG, which is a remarkably high proportion, even by the international standard [2].

LSG is considered a relatively safe BMS, but there is a small incidence of staple line leakage (referred to as “sleeve leakage”) ranging from 0.7% to 5%, which can be difficult to treat and may require reoperation as the only recourse in some cases [3]. In Japan, the reported incidence of sleeve leakage is lower, at 0.5% [4], which could be attributed to the successful introduction and dissemination of LSG under Japan’s national health insurance (NIH) system. Most cases of BMS in Japan have been performed by qualified and experienced laparoscopic surgeons at a limited number of specially accredited medical facilities. These accredited centers for BMS, approved by the government, are required to have a multidisciplinary team and hospital equipment that meet institutional requirements, as outlined in the guidelines issued by JSTO [5]. However, there have been instances where reoperation was necessary after multiple treatment interventions [6]. With an increase in the number of LSG procedures and surgeons performing them in the future, it is essential for surgeons to be familiar with the hallmarks and treatment options for sleeve leakage in advance. This study aimed to provide an overview of the pathogenesis of sleeve leakage and its treatments, ranging from less invasive methods to revision surgery.

## 2. Materials and Methods

This narrative review focused on peer-reviewed literature and used appropriate gray literature when required.

## 3. Features of Sleeve Leakage

### 3.1. Causes of Sleeve Leakage

Approximately 75–85% of sleeve leakages occur at the proximal third of the greater curvature staple line, which is a difficult area to treat and usually occurs on postoperative day 5 or later [7]. The upper part of the sleeved stomach, especially around the angle of His, has lower blood flow and thinner gastric walls than other areas of the stomach. Therefore, excessive resection of the Belsey’s fat pad near the angle of His, thermal damage to the stomach wall by energy devices, staple misalignment, and incisions into the abdominal esophagus can cause sleeve leakage [8]. In addition, the intragastric pressure of the sleeved stomach is higher than that of the normal anatomy. In cases of passage obstruction due to distal stenosis (especially at the gastric angle) or twisting, the intragastric pressure further increases, making sleeve leakage more likely [8].

### 3.2. Symptoms and Timing of Onset of Sleeve Leakage

Symptoms of sleeve leakage often include abdominal pain, back pain, fever of 38 °C or higher, and tachycardia (over 120 beats per minute) [9]. Sleeve leakage may occur within approximately 2 days after surgery if it is caused by technical issues or after 5 days or more following surgery if it is due to ischemia. Overall, it occurs in approximately 80% of patients within 2 weeks of surgery [10]. Since sleeve leakage might develop after discharge or even several months after the LSG, if symptoms are present, it should be considered a complication of the LSG and should be approached for diagnosis and treatment. Csendes et al. [11] classified the timing of sleeve leakage as early (postoperative days 1–3), intermediate (postoperative days 4–7), or late (postoperative days 8 or later), which are sometimes used in the treatment algorithm for sleeve leakage.

## 4. Diagnosis of Sleeve Leakage

When sleeve leakage is suspected, blood tests and simple computed tomography (CT) (or contrast-enhanced CT) should be performed immediately. In addition, the use of a water-soluble contrast medium (such as Gastrografin^®^) during a CT scan can be helpful in confirming the site of the leakage, as the detection of air bubbles or the extravasation of contrast medium outside the staple line may be indicative of sleeve leakage. Fluoroscopy with a water-soluble contrast agent can also be used to identify the leakage site. However, patients with obesity have thick abdominal walls and a clear image may not be produced, even with an undiluted contrast medium; therefore, care must be taken to avoid false negatives. The extent of the abscess cavity (size and spread) can also affect subsequent treatment strategies; therefore, contrast-enhanced CT scans are ideal if renal function allows [12]. If sleeve leakage cannot be definitively diagnosed by imaging, but the possibility of sleeve leakage cannot be ruled out based on the patient’s condition (especially if vital signs are unstable), diagnostic laparoscopy should be performed [13]. Patients with obesity can present difficulty in obtaining abdominal findings typical of peritonitis due to excess subcutaneous fat and low physical reserve. Therefore, it is crucial to remember that if complications are not promptly managed, a patient’s general condition can rapidly deteriorate.

## 5. Treatments of Sleeve Leakage

Owing to the low incidence of sleeve leakage, it is difficult to recommend a high-evidence-based treatment algorithm. The treatment strategy for leaks varies depending on the timing of leak occurrence, the size and spread of the abscess cavity, and the location and condition of the leak (orifice diameter, surrounding tissue conditions, etc.) [14]. In addition, there are limitations to the modalities that can be used for treatment, depending on the country and region. In this section, we discuss the treatment methods currently available in Japan for sleeve leakage and some noteworthy treatment methods.

Sleeve leakage tends to occur more often in the proximal third of the sleeved stomach, including near the angle of His, and can often be challenging to manage. This is likely due to the aggressive dissection of Belsey’s fat and the posterior attachments of the upper sleeve, which can result in a tenuous blood supply, thermal injury to the gastric wall from the use of ultrasonic devices, and a mismatch between staple height and tissue thickness. Patients with distal stenosis may be especially challenging to treat for proximal leaks because their gastric emptying impairment can lead to increased intraluminal pressure and decreased compliance of the gastric tube [9]. Therefore, we mainly focused on treating the sleeve leakage in the proximal third area. Leaks from other parts of the sleeved stomach, such as the middle or lower region, can be managed by adopting the treatment for anastomotic leaks in gastrointestinal surgery. This involves draining fluid collections, attempting to close the defect if feasible, and providing supportive interventions, such as antibiotics and/or nutritional support.

### Initial Treatments for Sleeve Leakage

The initial response should involve the administration of nil per os, an intravenous drip, and broad-spectrum antibiotics. Prompt consideration should be given to draining the leakage sites. Although CT-guided drainage is the preferred initial method, a transthoracic approach may be necessary because the abscess cavity is located below the left diaphragm and near the angle of His. Laparoscopic drainage is frequently used because of its ability to facilitate concurrent washout and primary repair of the leak site [14].

The timing of leakage and extent of abscess formation determine whether suturing is viable for closing the fistula. If the surrounding tissue is non-brittle, it may be possible to close the leak site with suturing. However, if the tissue is fragile, the area should be patched with the greater omentum, if possible, followed by appropriate drain placement. When a passage disturbance is caused by a stenosis or a twisting distal to the leak site, increased intragastric pressure can make it difficult for the leak site to heal. Therefore, suturing the gastric tube to the mesentery or abdominal wall during reoperation is advisable in preventing such complications [15]. Enteral nutrition has also been reported to accelerate leak healing. If delayed healing is expected, gastrostomy or enterostomy is recommended as the route of administration for enteral nutrition [14].

Observation of the leak site using intraoperative endoscopy is useful in determining subsequent treatment strategies. Therefore, during surgery where washing and drainage can be performed, intraoperative endoscopy should be performed to observe the leak site, if possible. However, performing endoscopic treatments such as clipping with OTSC^®^ (see below) or stent placement, as reported in other countries, at the same time may be difficult outside of highly specialized facilities due to the preparation of the devices.

## 6. Conservative Treatment following Initial Treatments

### 6.1. Kangaroo™ W-ED Tube (Figure 1)

The W-ED tube (Cardinal Health, Tokyo, Japan) is a medical device developed in Japan that is not currently available in other countries. Nonetheless, it is a highly appealing alternative treatment for patients undergoing gastrointestinal surgery who experience postoperative leakage. The W-ED tube has a double-tube structure, with three side holes at the tip of the tube that can be placed in the duodenum or jejunum and six side holes 40 cm from the tip that can be placed at the site of the leak, allowing for enteral nutrition, gastric decompression, and drainage simultaneously [16]. In the case of a pinhole leak without gastric tube stenosis after LSG, healing of the leak can be expected. At a cost of US$17, the W-ED tube can be considered a cost-effective option for placement during a leakage event. However, there is only one standard model with a 5.2 mm (16 Fr) tube outer diameter and 150 cm length; the tube is stiffer than a nasogastric tube of the same size, making it unsuitable for long-term placement in patients with severe nasal or pharyngeal discomfort or pain.

**Figure 1:**
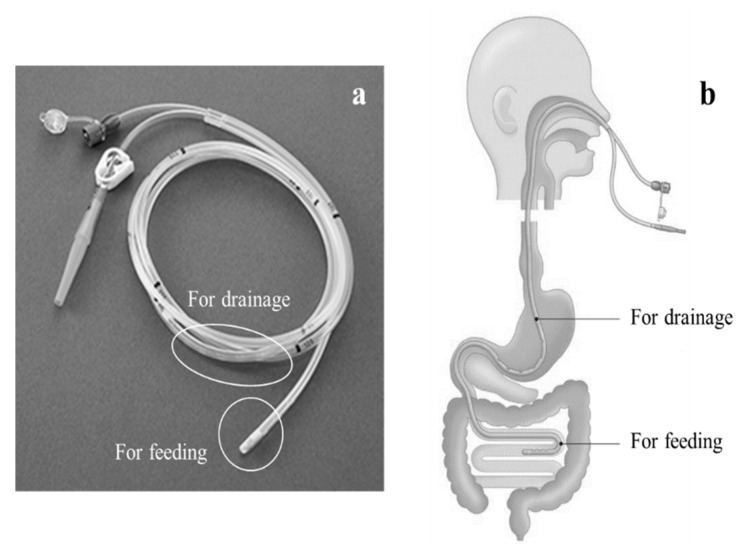
(**a**) Kangaroo™ W-ED Tube (Cardinal Health, Tokyo, Japan). (**b**) Schema for the dual use of drainage and nutrition through a single tube.

### 6.2. Over-The-Scope-Clip (OTSC^®^) (Figure 2)

Successful cases of endoscopic treatment of sleeve leakage using the OTSC^®^ (Ovesco, Tuebingen, Germany) have also been reported [9]. OTSC^®^ provides significantly more strength and better tissue capture compared to conventional endoscopic hemostatic clips. In addition, because blood flow is maintained through the inter-prong space of the clip, the OTSC^®^ can prevent tissue necrosis and aid wound healing. As necessary, twin graspers and anchor forceps are used as auxiliary forceps [17]. Successful endoscopic closure of the leak using the OTSC^®^ device depends on its ability to suction the relatively healthy tissue surrounding the leak into the cylindrical cap. Because position correction or clip retrieval after release is difficult and the device cannot be easily repositioned, an experienced endoscopist is necessary to handle the device. The OTSC^®^ is also approved in Japan as a treatment device for fistulas in the gastrointestinal tract. The OTSC system costs US$600 for one set (one clip) or US$600 and US$695 if an anchor and twin grasper are used, respectively.

**Figure 2:**
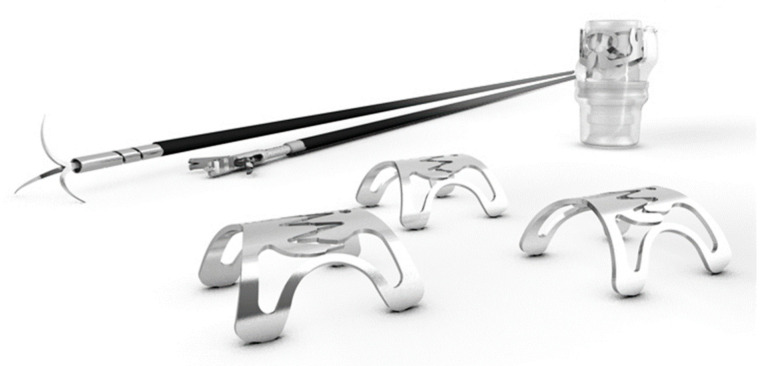
Over-The-Scope Clip (OTSC^®^) (Ovesco, Tuebingen, Germany). Clipping is performed after aspirating the fistula opening in the applicator cap, as in endoscopic varicocele ligation. An anchor or twin grasper can be used as an adjunctive tool to ensure lesion capture.

### 6.3. Endoscopic Balloon Dilation

**Figure 3:**
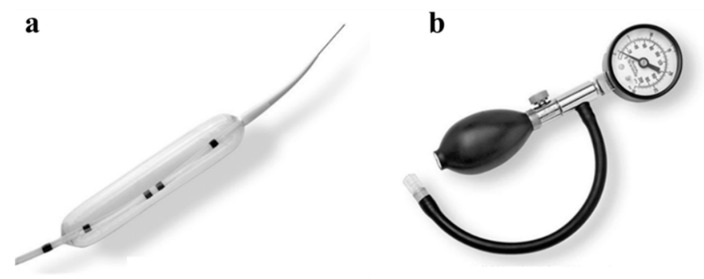
(**a**) Rigiflex™ II Single-Use Achalasia Balloon Dilators. (**b**) Achalasia Pneumatic Hand Pump and Monitor. (Boston Scientific, Marlborough, MA, USA).

To achieve successful leak closure, it is imperative to prevent disturbances to the distal passage beyond the leakage site. Although the lack of issues encountered during endoscopy with the passage of the scope may not necessarily guarantee the absence of passage obstruction, identifying poor contrast medium outflow through fluoroscopy can serve as a useful indicator of passage obstruction. For anastomotic stenosis after gastrointestinal surgery, balloon catheters <18 mm in diameter used in the through-the-scope technique are insufficient for dilating gastric strictures after LSG. In many cases, a balloon with a diameter >30 mm, such as that used for achalasia, is recommended (Figure 3). It is worth attempting the endoscopic balloon dilation procedure a few more times, even if the first or second attempts are unsuccessful, as the third dilation may sometimes result in stenosis release [18]. However, given the need for multiple treatments, the cost of using a single-use achalasia balloon (Figure 3a) and pneumatic hand pump (Figure 3b) may be a barrier. Each balloon costs US$990, while each hand pump costs US$245. Additionally, achalasia balloon dilation involves passing a catheter through the stenosis along a guidewire under fluoroscopic guidance, which can be risky in cases of severe gastric tube tortuosity.

### 6.4. Stent Placement

**Figure 4:**
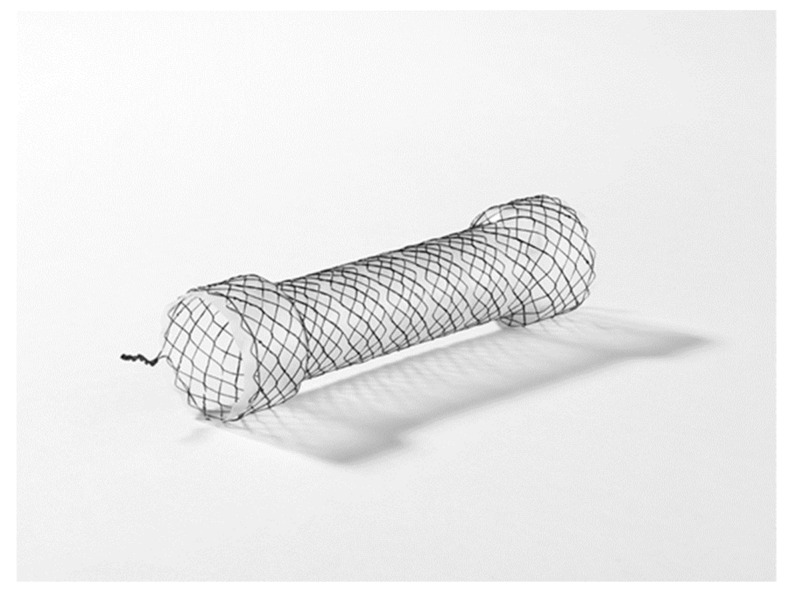
HANAROSTENT^®^ (M.I. Tech., Pyeongtaek-si, Republic of Korea). The stent consists of a membrane, gold radiopaque markers, and a retrievable lasso attached to its proximal end.

Self-expandable removable stent treatment has a high success rate of up to 80% for leak closure. Endoscopic stenting is considered one of the standard treatments for sleeve leakage [14]. Stenting offers the advantage of simultaneously covering the leak site and releasing the gastric stricture, and we believe that leaks near the esophagogastric junction are the best indications for stenting [19]. In clinical practice, esophageal stents are often used to treat sleeve leakage. In Japan, full-coverage esophageal stents available include HANAROSTENT^®^ (M.I. Tech., Pyeongtaek-si, Republic of Korea; Figure 4) for the over-the-wire method and Niti-S^®^ (Taewoong Medical, Gimpo-si, Republic of Korea) for the through-the-scope method. The length of the stent should be determined based on the location of the sleeve leakage and the distance to the stenosis. Although some reports suggest the effectiveness of long stents [20], it should be noted that stents that are too long may obstruct the outflow tract or cause erosion/ulceration due to contact between the stent tip and the gastric wall.

The standard duration of stent placement was 6–8 weeks. The problems associated with stents included early removal due to stent migration, pain, nausea, discomfort caused by stent placement, and uncertainty regarding their efficacy when the leak is incompletely covered [9]. In the Japanese NHI system, esophageal stents are approved solely for treating strictures caused by esophageal cancer and not for managing sleeve leakage. Consequently, the cost of using them, which amounts to US$960, is not covered by the NHI. Moreover, prior permission from the hospital’s ethics committee is generally required for their use as treatment options. This can make it challenging to consider them as viable treatment options.

### 6.5. Percutaneous Transesophageal Gastro-Tubing (PTEG)

As previously reported, PTEG can serve the dual purposes of splinting the leakage site and providing jejunal nutritional access while managing sleeve leakage near the esophagogastric junction [21]. Compared to a nasogastric tube, PTEG causes less pain and discomfort and is more beneficial for patients who require long-term tube use.

**Figure 5:**
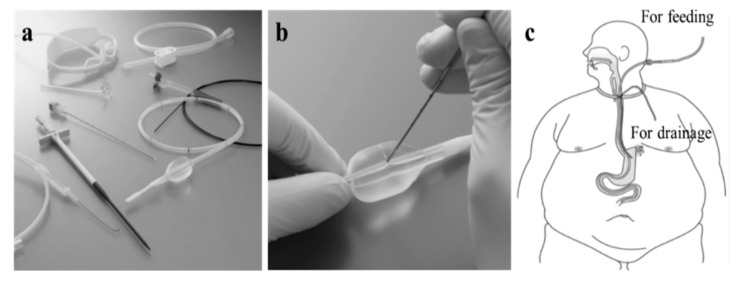
(**a**) PTEG kit (SB-KAWASUMI LABORATORIES, INC., Akita, Japan). (**b**) The rupture-free balloon is not ruptured by needle puncture. (**c**) Image of the double percutaneous transesophageal gastro-tubing.

The PTEG kit (SB-KAWASUMI LABORATORIES, INC., Akita, Japan) is commercially available for US$500 and includes ready-to-use devices, such as a needle with a sheath, rupture-free balloon (RFB), dilator, guidewires, and long placement tube (Figure 5a,b). Furthermore, an additional indwelling tube through the same opening as the primary PTEG (double PTEG) simultaneously enables separate enteral nutrition access and gastric drainage (Figure 5c). This technique is described in a previously reported video [22].

The PTEG was initially performed in malnourished patients who experienced difficulties with oral feeding under local anesthesia and intravenous sedation [23]. However, in patients with obesity, dilation of the RFB in the cervical esophagus and added pressure on the neck with an ultrasonic transducer can easily trigger a vomiting reflex. Therefore, it is recommended to use general anesthesia to ensure stability during PTEG for patients with obesity [21].

Performing PTEG (or double PTEG) in patients with obesity who require BMS can be challenging; however, it could be a viable treatment option prior to pursuing more invasive measures for post-BMS complications. This is particularly relevant when other conservative treatments have proven ineffective.

### 6.6. Endoscopic Vacuum Therapy (EVAC)

**Figure 6:**
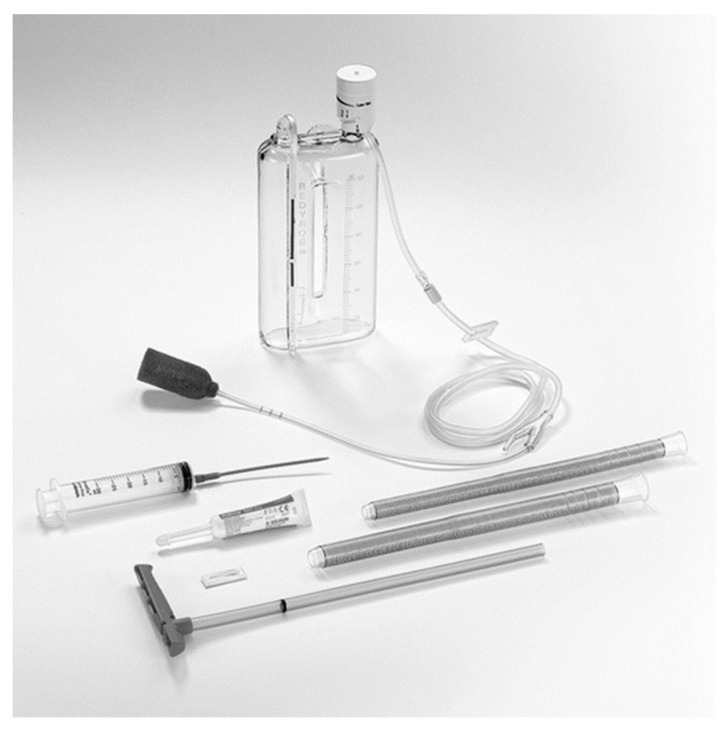
Endo-SPONGE^®^ kit including open-pore sponge drain, two silicon overtubes, the sponge pusher, and the irrigation set (B. Braun, Meslungen, Germany).

In recent years, EVAC has been used to treat leaks following LSG [24]. EVAC is a negative pressure closure therapy that involves the endoscopic placement of a porous polyurethane sponge in the abscess cavity at the leak site. Although it is unavailable in Japan, Endo-SPONGE^®^ (B. Braun, Meslungen, Germany; Figure 6) has already been commercialized and used in other countries. Each set of kits is priced at US$220. EVAC is expected to exert drainage effects, increase local blood flow, and promote granulation tissue formation. While it may be inconvenient to keep the nasogastric tube in place for continuous suction and change the sponge every 3–5 days, based on the size of the abscess cavity, this treatment typically results in a relatively rapid and high healing success rate (within approximately 2 weeks) [24].

Indications and characteristics of the devices for sleeve leakage are summarized in Table 1.

## 7. Reoperation (Revisional Surgery)

When other conservative treatment methods are ineffective, revisional surgery is considered the final option for sleeve leakage. However, there is no consensus regarding when this should be done. Based on international literature, leaks tend to heal in approximately 8.8 weeks with successful conservative treatment [14]. However, if a leak persists for over 12 weeks, it is considered chronic and may not respond to conservative treatment [3]. Therefore, revisional surgery should be considered. Prolonged conservative treatment not only protracts the patient’s psychological and physical suffering, but also results in persistent leakage of gastric juice, leading to serious complications, such as gastrobronchial fistula formation and perigastritis [25]. Perigastritis can cause severe adhesions around the stomach, thereby increasing the risk of reoperation. Therefore, the timing is crucial and should not be overlooked.

### 7.1. Fistulo-Jejunostomy (Figure 7a)

When adhesions surrounding the leak site are not severe and can be dissected and the opening of the leak is visible, a technique such as Roux-en-Y reconstruction can be employed. This approach involves repairing the defect using a Roux limb patch. The major advantage of this technique is that it avoids a total or proximal gastrectomy. After an anastomosis is formed between the fistula orifice and the jejunum, in which a small opening has been created, the Roux limb can serve as a channel for saliva and food to exit, thereby improving sleeve leakage. Inserting an additional nasogastric tube into the Roux limb can assist in keeping the anastomosis open while enabling enteral feeding, which can aid in a more rapid improvement of the patient’s condition [26].

**Figure 7:**
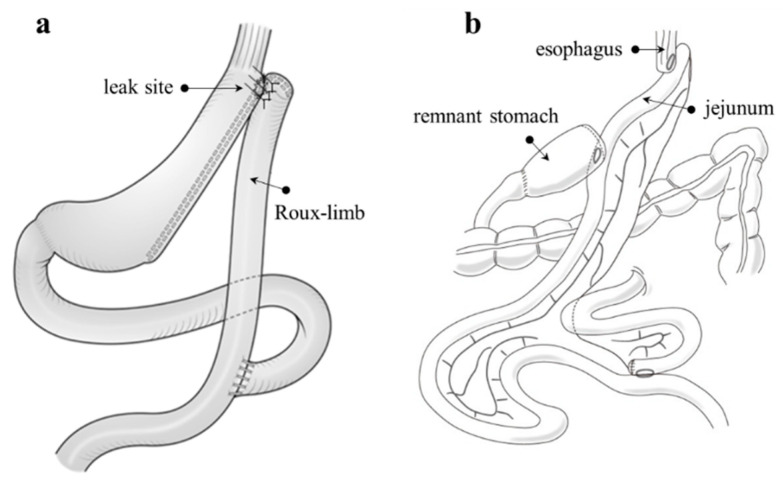
(**a**) The Roux limb is patched largely around the fistula orifice. (**b**) Schematic illustration of proximal gastrectomy with double tract reconstruction. The term ‘double tract’ refers to the bi-directional flow of food through the remnant stomach and jejunum after reconstruction, which involves three anastomoses: esophagojejunostomy, gastrojejunostomy, and jejunojejunostomy.

### 7.2. Total or Proximal Gastrectomy

If the site of a leak cannot be identified or if the surrounding tissue is too fragile for anastomosis, it may be necessary to perform a total or proximal gastrectomy that includes the leak site, followed by esophagojejunostomy. Roux-en-Y reconstruction following total gastrectomy is considered to pose a lower risk of leakage due to the establishment of an anastomosis between the parts of the healthy digestive tract (esophagojejunostomy and jejunojejunostomy). However, the problem of a postgastrectomy syndrome, particularly dumping syndrome, remains. Proximal gastrectomy with double-tract reconstruction is also a viable option (Figure 7b), although it carries similar risks and requires more anastomotic sites than total gastrectomy. With this procedure, the distal side of the stomach is retained, which can help reduce the incidence of pernicious anemia and allow standard endoscopic access to the biliary tract after surgery [6].

## 8. Discussion

Sleeve leakage is a serious complication that may occur after LSG, but its risk can be reduced using various techniques and approaches. These include paying attention to the minimum necessary dissection, avoiding ischemic complications, staying away from the gastroesophageal junction, and using steady compression on the staple device. It is also important to avoid narrowing the sleeve at the incisura angularis in order to prevent partial obstruction and an increase in the leak rate. While staple line reinforcement, fibrin sealants, and bougie size are all potential strategies to decrease the leakage rate, further research is needed to determine their effectiveness [8,9]. Complying with every post-operative instruction, particularly the content, amount, and manner of eating, can also aid in preventing sleeve leakage. After LSG, surgeons must remain highly suspicious of symptoms like abdominal pain, fever, tachycardia, and breathing difficulty to detect the leak early and avoid life-threatening complications.

This study focused on the symptoms, diagnosis, conservative treatment, and surgical treatment of sleeve leakage. As the demand for LSG continues to increase, the number of procedures is expected to increase [1]. However, with the ability to minimize the risks of surgery, sleeve leaks are occurring less frequently and interest in their treatment tends to wane. Nonetheless, once sleeve leakage occurs, treatment can be challenging because of anatomical issues after surgery, such as the occurrence of ischemic areas and increased intragastric pressure. It is essential to keep in mind that the mortality from sleeve leaks is possible [27].

Although several reports have proposed diagnostic and treatment algorithms for sleeve leakage [3,14], the low number of cases of this complication makes it challenging to generate high-quality, evidence-based reports. Personalized treatment approaches are necessary for managing sleeve leakage; however, several factors, including the timing and location of the leakage, its size, the shape of the sleeved stomach, the patient’s tolerance for treatment and social background, and differences in realistic treatment options across regions and hospitals, must be considered when deciding treatment strategies.

As mentioned earlier, most surgeons would agree on the initial treatment for sleeve leakage. However, strategies following initial treatment should be based on effective and minimally invasive treatment options. Medical costs should also be considered. Careful consideration is necessary when determining the timing of treatment evaluation because continuing ineffective treatment may negatively impact patient outcomes.

Surgery is the primary treatment option for sleeve leakage [28]. However, the patient’s overall condition should be stabilized as much as possible before surgery. Therefore, local drainage and enteral nutrition should be prioritized during all stages of treatment. There is no established timing for revisional surgery; however, if conservative treatments fail, decisions should be made before the local chronic inflammation progresses to reduce the difficulty of surgery. In revisional surgery, the surgical risk is higher than that during the initial surgery due to tissue hardening, edema, adhesion, and increased bleeding propensity. Thus, it is desirable to have expertise not only in primary BMS, but also in troubleshooting and reoperation in gastrointestinal surgery.

## 9. Conclusions

It is important to acknowledge that leaks following LSG present distinct challenges compared to other gastrointestinal surgical complications. Although some of the treatments described in this paper are not covered by the NHI in Japan and some products originating in Japan are not distributed in other countries, it is important to know and share the various treatment options of sleeve leakage to prepare for possible situations that may arise.

## Figures and Tables

**Table 1:** Indications, advantages, and disadvantages of less invasive treatment methods for sleeve leakage.

Treatment	Suggested Good Indications	Advantages	Disadvantages
W-ED tube®	▪ Pinhole leak without gastric tube stenosis	▪ Simultaneous enteral nutrition, gastric decompression, and drainage ▪ Low cost	▪ Stiffer than a nasogastric tube, unsuitable for long-term placement ▪ Not currently available overseas
OTSC®	▪ Leak site allows suctioning surrounding healthy tissue into the applicator cap of OTSC	▪ Provides more strength and better tissue capture than conventional clips ▪ Maintains blood flow and aids wound healing through inter-prong space	▪ Difficult to reposition or retrieve clip after release ▪ Requires an experienced endoscopist
Endoscopic balloon dilation	▪ Leak with gastric tube stricture	▪ Can be attempted multiple times	▪ High cost with single-use achalasia balloon and pneumatic hand pump ▪ Risky in cases of severe gastric tube tortuosity ▪ Not a fundamental treatment for leaks.
Stent	▪ Standard treatment for leak	▪ Simultaneously covers the leak site and corrects gastric tube stenosis and axis deviation ▪ High success rate of up to 80% for leak closure	▪ May cause unendurable symptoms, such as nausea, vomiting, and retrosternal discomfort ▪ Concerns of migration and insufficient sealing
PTEG	▪ Leak requiring long-term tube placement	▪ Splinting the leakage site and providing jejunal nutritional access ▪ Double PTEG also available ▪ Causes less pain and discomfort compared to nasogastric tube	▪ Challenging to perform, probably requires general anesthesia to ensure stability
EVAC	▪ Any kinds of leak	▪ Expected to exert drainage effects, increase local blood flow, and promote granulation tissue formation ▪ Rapid and high healing success rate within approximately 2 weeks	▪ Inconvenient to keep the nasogastric tube in place for continuous suction and change the sponge every 3–5 days based on the size of the abscess cavity

OTSC, Over-The-Scope-Clip; PTEG, Percutaneous transesophageal gastro-tubing, EVAC, Endoscopic Vacuum Therapy.

## Data Availability

Not applicable.
